# Supplementary material for: Preconception Lifestyle and Cardiovascular Health in the Offspring of Overweight and Obese Women
Source: Nutrients. 2019 Oct 14;11(10):2446. doi: 10.3390/nu11102446 (PMC6835651; doi:10.3390/nu11102446)
Supplement: Supplementary file 1 [file nutrients-11-02446-s001.zip › Table S2.pdf]

Table S2. Linear regression analysis of the preconception food score (0-5 points; the higher the more healthy) and offspring cardiovascular health at age 3-6 years.

Preconception food score (0-5 points)			
**Offspring health**	**N**	**β (95% C.I.) unadjusted**	**P-value**
BMI (Z-Score)	43	0.02 (-0.27; 0.30)	0.90
Waist:height (ratio)	43	-0.003 (-0.01; 0.01)	0.42
SBP (Z-Score)	41	-0.02 (-0.18; 0.13)	0.76
DBP (Z-Score)	41	-0.12 (-0.27; 0.03)	0.10
Fat mass (%)	40	1.37 (-1.12; 3.87)	0.27
Fat free mass (kg)	40	0.30 (-0.36; 0.96)	0.36
Pulse wave velocity (m/s)	32	0.21 (-0.13; 0.54)	0.22

BMI = body mass index; SBP = systolic blood pressure; DBP = diastolic blood pressure.
