# Supplementary material for: Preconception Lifestyle and Cardiovascular Health in the Offspring of Overweight and Obese Women
Source: Nutrients. 2019 Oct 14;11(10):2446. doi: 10.3390/nu11102446 (PMC6835651; doi:10.3390/nu11102446)
Supplement: Supplementary file 1 [file nutrients-11-02446-s001.zip › Table S1.pdf]

Table S1. Women and offspring characteristics of the participants (N = 46) vs. the non-participants (N = 259).

Women's characteristics					
	**N**	**Participants**	**N**	**Non-participants**	**P-value**
Age at pregnancy (years; mean; SD)	46	30.1 (3.9)	254	29.8 (4.3)	0.69
Caucasian (yes; N; %)	46	44 (95.7)	259	227 (87.6)	0.11
Education level (N; %)	44		246		0.29
No education or primary school		0		10 (4.1)	
Secondary school		10 (22.7)		59 (24.0)	
Intermediate vocational education		26 (59.1)		115 (46.7)	
Higher vocational education and university		8 (18.2)		62 (25.2)	
Nulliparous (yes; N; %)	46	34 (73.9)	258	207 (80.2)	0.33
Pre-pregnancy BMI (kg/m^2; mean; SD)	46	35.3 (3.4)	246	35.1 (3.8)	0.77
Gestational diabetes (yes; N; %)	46	11 (23.9)	252	44 (17.5)	0.30
Smoking at randomization (yes; N; %)	46	8 (17.4)	255	56 (22.0)	0.49
Mode of conception (N; %)	46		255		0.77
Spontaneous		18 (39.1)		97 (38.0)	
Ovulation induction		16 (34.8)		78 (30.6)	
Intra Uterine Insemination		7 (15.2)		37 (14.5)	
IVF/ICSI/CRYO		5 (10.9)		43 (16.9)	
PCOS (yes; N; %)	46	20 (43.5)	258	96 (37.2)	0.42
Vegetable intake (gram/day)	45	121.4 (92.9; 164.3)	228	114.3 (85.7; 171.4)	0.93
Fruit intake (gram/day)	45	100.0 (57.1; 142.9)	228	100.0 (57.1; 171.4)	0.85
Sugary drinks (glasses/day)	43	0.75 (0.15; 1.61)	214	0.65 (0.20; 1.84)	0.98
Savoury snacks (handful/week)	43	1.8 (0.8; 4.4)	219	1.9 (0.8; 5.0)	0.20
Sweet snacks (portion/week)	43	2.5 (0.9; 5.0)	220	2.5 (0.9; 5.0)	0.95
Total moderate-vigorous physical activity (min/week)	45	480.0 (165.0; 695.0)	228	402.5 (150.0; 960.8)	0.94
**Offspring's characteristics**					
	**N**	**Participants**	**N**	**Non-participants**	**P-value**
Sex (boys; N; %)	46	22 (47.8)	253	133 (52.6)	0.55
Birth weight (grams; mean; SD)	46	3497.4 (507.2)	253	3390.9 (584.8)	0.25
Gestational age at birth (weeks; mean; SD)	46	39.1 (1.7)	254	39.0 (2.0)	0.79
Exclusively breastfed (months; median; IQR)	42	0.0 (0.0; 2.0)	48	0.0 (0.0; 1.0)	0.49
Age of the child at time of assessment (years; mean; SD)	46	4.7 (1.0)	N.A.	N.A.	N.A.

SD = standard deviation; BMI = Body Mass Index; IVF = In Vitro Fertilisation; ICSI = Intracytoplasmic Sperm Injection; CRYO = Cryotherapy; min/week = minutes/week; PCOS = polycystic ovary syndrome.
